# A clinical model to predict the risk of synchronous bone metastasis in newly diagnosed colorectal cancer: a population-based study

**DOI:** 10.1186/s12885-019-5912-x

**Published:** 2019-07-17

**Authors:** Xu Guan, Chen-xi Ma, Ji-chuan Quan, Shuai Li, Zhi-xun Zhao, Hai-peng Chen, Ming Yang, Zheng Liu, Zheng Jiang, Xi-shan Wang

**Affiliations:** 1 Department of Colorectal Surgery, National Cancer Center/National Clinical Research Center for Cancer/Cancer Hospital, Chinese Academy of Medical Sciences and Peking Union Medical College, Bejing, China; 2 Department of Anesthesiology, National Cancer Center/National Clinical Research Center for Cancer/Cancer Hospital, Chinese Academy of Medical Sciences and Peking Union Medical College, Bejing, China

**Keywords:** Colorectal cancer, Bone metastasis, Risk model, Risk factors, The surveillance, epidemiology, and end results database

## Abstract

**Background:**

The early detection of synchronous bone metastasis (BM) in newly diagnosed colorectal cancer (CRC) affects its initial management and prognosis. A clinical model to individually predict the risk of developing BM would be attractive in current clinical practice.

**Methods:**

A total of 55,869 CRC patients were identified from Surveillance, Epidemiology, and End Results (SEER) database, of whom 317 patients were diagnosed with synchronous BM. Risk factors for BM in CRC patients was identified using multivariable logistic regression. A weighted scoring system was built with beta-coefficients (*P* < 0.05). A random sample of 75% of the CRC patients was used to establish the risk model, and the remaining 25% was used to validate its accuracy of this model. The performance of risk model was estimated by receiver operating curve (ROC) analysis.

**Results:**

The risk model consisted of 8 risk factors including rectal cancer, poorly-undifferentiation, signet-ring cell carcinoma, CEA positive, lymph node metastasis, brain metastasis, liver metastasis and lung metastasis. The areas under the receiver operating curve (AUROC) were 0.903 and 0.889 in the development and validation cohort. Patients with scores from 0 to 4 points had about 0.1% risk of developing BM, and the risk increased to about 30% in patients with scores ≥15 points.

**Conclusions:**

This clinical risk model is accurate enough to identify the CRC patients with high risk of synchronous BM and to further provide more individualized clinical decision.

## Background

Colorectal cancer (CRC) is the most commom cancer and the second most common cancer cause of death wordwide, leading to more than 1.8 million new cases and 881 thousand deaths in 2018 [1]. CRC is most likely to metastasize to liver, followed by lungs and peritoneal cavity, yet rarely to bone [2]. The incidence of bone metastasis (BM) among CRC patients has been reported to be 6.0–10.4% with higher rate of 8.6–23.7% in the autopsy results [3]. Nevertheless, the BM incidence rate from CRC has increased in recent years [4], and it is usually diagnosed at the advanced stages with a 5-year survival rate less than 5% [5].

In newly diagnosed CRC patients, systematic body screening including liver and lung examinations is routinely recommended by current guideline [6]. However, because of low incidence of BM, body imaging regarding to synchronous BM is mostly ignored during the primary diagnoses of CRC. And patients are often advised to perform radionuclide bone imaging or PET-CT only when they have suspicious symptoms of skeletal-related events (SREs), which are the concomitant complications of BM and exist high incidence within 1 year after BM [7]. At this time, the CRC are likely to have reached advanced stage or multiple metastases have occurred, thus the best chance of treatment for CRC patients will be missed [8]. In addition, the occurrence of SREs, including bone pain, pathological fracture, possible radiotherapy, spinal cord compression, fatal hypercalcemia could further lower the quality of life and survival of patients [9].

The identification of clinical and tumor factors related to synchronous BM will play an important role in the early detection of synchronous BM among newly diagnosed CRC patients. A clinical risk model of predicting BM appears to be a helpful way to clarify how likely a patient would suffer from BM. Several studies have identified the risk factors associated with BM [10, 11], however most of models tend to predict the risk of metachronous BM which is diagnosed after curative resection of CRC [12, 13]. To date, there is no statistical risk model has been developed for predicting the probability of synchronous BM at primary CRC diagnosis.

Thus, the aim of our study was to utilize the population-based data to identify the risk factors associated with high risk of synchronous BM and to develop a risk model to predict the likelihood of synchronous BM for newly diagnosed CRC patients, which could potentially alert clinicians to detect BM promptly in patients with CRC and further take appropriate measures to avoid the incidence of SREs.

## Methods

### Study population

**Fig. 1:**
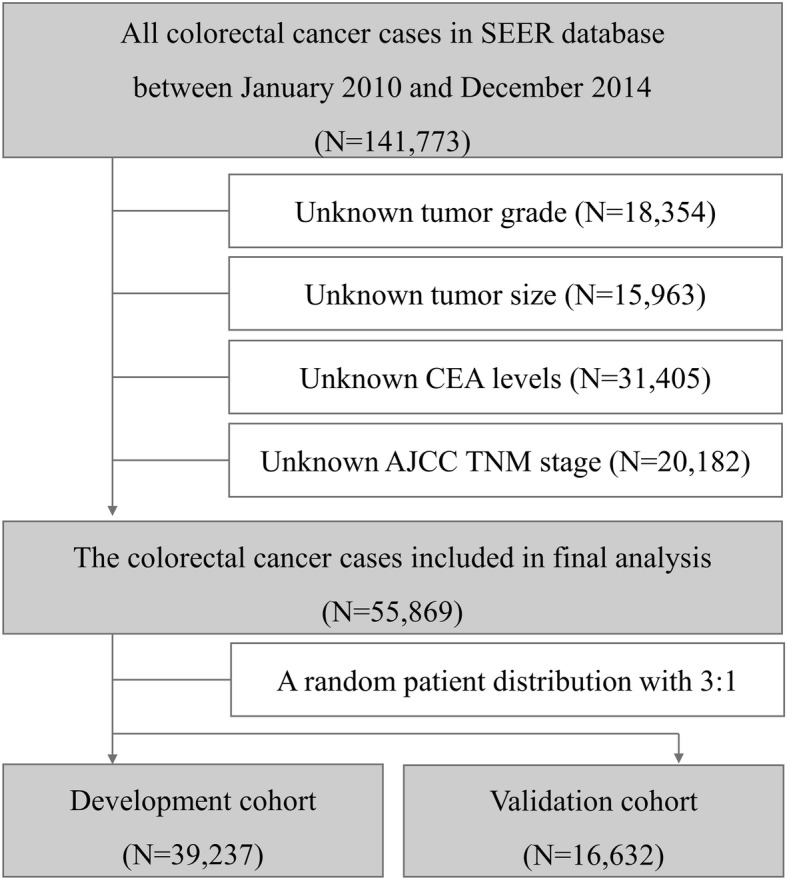
Analytical cohort and exclusion criteria

The newly diagnosed CRC cases were extracted from the Surveillance, Epidemiology, and End Results (SEER) database between January 2010 and December 2014. The SEER database includes the information with regard to cancer incidence, survival outcome and treatment strategy from 17 population-based cancer registries, representing 28% of US population [14]. All CRC patients included in this study were definitively diagnosed by pathological examination, and BM were diagnosed using imaging examination and/or pathological examination. Initially, 141,773 patients were identified who was diagnosed with CRC in the database. After excluding 85,904 cases who were not eligible, we finally collected 55,869 patients at primary diagnosed CRC in AJCC TNM stage I to IV (Fig. 1). Patient demographics and tumor variables were collected. The demographic variables included age at diagnosis, gender, race. The tumor variables included the primary tumor location, degree of tumor differentiation, tumor size, pathological type, carcinoembryonic antigen (CEA) levels, T stage, N stage and the extent of distant metastatic site involving bone, brain, liver and lung at primary diagnosis of CRC in the SEER database. Since the data collected form the SEER database were anonymized and de-identified prior to release, they do not require informed patient consent in our study. This study was approved by the Ethics Committee of National Cancer Center/National Clinical Research Center for Cancer/Cancer Hospital, Chinese Academy of Medical Sciences and Peking Union Medical College institutional review board. All methods were performed in accordance with relevant guidelines of the SEER database.

### Statistical analysis

The patients were randomly divided into development cohort and validation cohort with a ratio of 3:1. In development cohort, the clinical and tumor variables between patients with and without BM were compared by using Spearman’s rank correlation coefficient. Then, variables that associated with BM (*P* < 0.05) were included in a multivariable logistic regression model. Risk factors for BM in CRC patients was identified from the statistically significant variables in the multivariable model. The extent of model discrimination was further estimated by calculating the area under the receiver operating characteristic curve (AUROC). All statistical analysis was performed with SPSS version 25.0 for Mac. It is considered as statistically significant when *P* < 0.05.

### Prediction of synchronous BM

The beta coefficients (β) were calculated in the multivariable model to develop a weighted point system to individually predict the risk of synchronous BM for newly diagnosed CRC patients. Individual patient scores were calculated by summing the score of each significant risk factor. The insignificant risk factors (*P* ≥ 0.05) received 0 point. Variables with β > 0 and < 0.5 received 1 point; those with β ≥ 0.5 and < 1 received 2 points; those with β ≥ 1 and < 1.5 received 3 points; those with β ≥ 1.5 and < 2 received 4 points; those β ≥ 2 and < 2.5 received 5 points and those β ≥ 2.5 and < 3 received 6 points. The risk model was created based on 75% of the CRC patient in the development cohort and validated on the remaining 25% of the patients in the validation cohort. Observed and predicted rates of BM for each point value were calculated. Risk stratification of BM was further performed based on the risk scores.

## Results

### Patient characteristics

A total of 55,869 patients diagnosed with CRC were ultimately included in the final analysis, of whom 317 patients were diagnosed with BM, accounting for 0.57% of all patients. Among these 317 patients, 61 patients were diagnosed with solitary BM, accounting for 19.2% of BM patients. The comparisons of clinical and tumor characteristics between all patients with BM and patients without BM were shown in Table 1. Finally, 208 patients with BM (0.53%) were included in the development cohort (*n* = 39,237), while 109 (0.66%) in the validation cohort (*n* = 16,632), which had no significant difference (*P* = 0.072). In the development cohort, variables including age at diagnosis, tumor location, tumor grade, tumor size, histological type, CEA levels, T stage, N stage, brain metastasis, liver metastasis and lung metastasis were associated with synchronous BM at the primary diagnosis of CRC, with *P* < 0.05. The details were shown in Table 2.

**Table 1:** The clinical and tumor characteristics between patients with bone metastasis and patients without bone metastasis

Characteristic	All patients (*N* = 55,869)	Patients without BM (*N* = 55,552)	Patients with BM (*N* = 317)	BM rate (%)
Age at diagnosis (years)				
< 60	19,665 (35.2%)	19,529 (35.2%)	136 (42.9%)	0.69
≥ 60	36,204 (64.8%)	36,023 (64.8%)	181 (57.1%)	0.50
Sex				
Female	26,804 (48.0%)	26,674 (48.0%)	130 (41.0%)	0.49
Male	29,065 (52.0%)	28,878 (52.0%)	187 (59.0%)	0.64
Tumor location				
Colon	45,414 (81.3%)	45,176 (81.3%)	238 (75.1%)	0.52
Rectum	10,455 (18.7%)	10,376 (18.7%)	79 (24.9%)	0.76
Grade				
Well-Moderate differentiation	44,840 (80.2%)	44,639 (80.4%)	201 (63.4%)	0.45
Poorly-Undifferentiation	11,029 (19.8%)	10,913 (19.6%)	116 (36.6%)	1.05
Histological type				
Adenocarcinoma	50,201 (89.8%)	49,929 (89.9%)	272 (85.8%)	0.54
Mucinous adenocarcinoma	4,543 (8.1%)	4,521 (8.1%)	22 (6.9%)	0.48
Signet-ring cell carcinoma	1,125 (2.1%)	1,102 (2.0%)	23 (7.3%)	2.04
CEA				
(−)	31,446 (56.3%)	31,386 (56.5%)	60 (18.9%)	0.19
(+)	24,423 (43.7%)	24,166 (43.5%)	257 (81.1%)	1.05
Tumor size (cm)				
< 2	5,012 (9.0%)	5,001 (9.0%)	11 (3.5%)	0.22
2–5	26,001 (46.5%)	25,882 (46.6%)	119 (37.5%)	0.46
≥ 5	24,856 (44.5%)	24,669 (44.4%)	187 (59.0%)	0.75
AJCC TNM stage				
I	10,209 (18.3%)	10,209 (18.4%)	0	–
II	17,151 (30.7%)	17,151 (30.9%)	0	–
III	19,322 (34.6%)	19,322 (34.8%)	0	–
IV	9,187 (16.4%)	8,870 (15.9%)	317 (100%)	3.45
AJCC T stage				
T1/T2	13,363 (23.9%)	13,320 (24.0%)	43 (13.6%)	0.32
T3/T4	42,506 (76.1%)	42,232 (76.0%)	274 (86.4%)	0.64
AJCC N stage				
N0	29,312 (52.5%)	29,242 (52.6%)	70 (22.1%)	0.24
N1/N2	26,557 (47.5%)	26,310 (47.4%)	247 (77.9%)	0.93
AJCC M stage				
M0	46,682 (83.6%)	46,682 (84.0%)	0	–
M1	9,187 (16.4%)	8,870 (16.0%)	317 (100%)	3.45
Extraosseous metastasis				
No	48,735 (87.2%)	48,674 (87.6%)	61 (19.2%)	0.13
Yes	7,134 (12.8%)	6,878 (12.4%)	256 (80.8%)	3.59
Brain metastasis				
No	55,783 (99.8%)	55,480 (99.9%)	303 (95.6%)	0.54
Yes	86 (0.2%)	72 (0.1%)	14 (4.4%)	16.28
Liver metastasis				
No	49,332 (88.3%)	49,237 (88.6%)	95 (30.0%)	0.19
Yes	6,537 (11.7%)	6,315 (11.4%)	222 (70.0%)	3.40
Lung metastasis				
No	54,215 (97.0%)	54,027 (97.3%)	188 (59.3%)	0.35
Yes	1,654 (3.0%)	1,525 (2.7%)	129 (40.7%)	7.80

*N* Number, *BM* Bone Metastasis

**Table 2:** The clinical and tumor characteristics between patients with bone metastasis and patients without bone metastasis in development cohort

Characteristic	Patients without BM (*N* = 39,029)	Patients with BM (*N* = 208)	*P* value
Age at diagnosis (years)			0.004
< 60	13,582 (34.8%)	92 (44.2%)	
≥ 60	25,447 (65.2%)	116 (55.8%)	
Sex			0.083
Female	18,671 (47.8%)	87 (41.8%)	
Male	20,358 (52.2%)	121 (58.2%)	
Tumor location			0.002
Colon	31,776 (81.4%)	152 (73.1%)	
Rectum	7,253 (18.6%)	56 (26.9%)	
Grade			0.000
Well-Moderate differentiation	31,361 (80.4%)	134 (64.4%)	
Poorly-Undifferentiation	7,668 (19.6%)	74 (35.6%)	
Histological type			0.000
Adenocarcinoma	35,131 (90.0%)	172 (82.6%)	
Mucinous adenocarcinoma	3,122 (8.0%)	18 (8.7%)	
Signet-ring cell carcinoma	776 (2.0%)	18 (8.7%)	
CEA			0.000
(−)	22,016 (56.4%)	39 (18.7%)	
(+)	17,013 (43.6%)	169 (81.3%)	
Tumor size (cm)			0.000
< 2	3,519 (9.0%)	10 (4.8%)	
2–5	18,211 (46.7%)	76 (36.5%)	
≥ 5	17,299 (44.3%)	122 (58.7%)	
AJCC T stage			0.000
T1/T2	9,346 (23.9%)	27 (13.0%)	
T3/T4	29,683 (76.1%)	181 (87.0%)	
AJCC N stage			0.000
N0	20,560 (52.7%)	48 (23.1%)	
N1/N2	18,469 (47.3%)	160 (76.9%)	
Brain metastasis			0.000
No	38,984 (99.9%)	199 (95.7%)	
Yes	45 (0.1%)	9 (4.3%)	
Liver metastasis			0.000
No	34,633 (88.7%)	62 (29.8%)	
Yes	4,396 (11.3%)	146 (70.2%)	
Lung metastasis			0.000
No	38,000 (97.4%)	128 (61.5%)	
Yes	1,029 (2.6%)	80 (38.5%)	

*N* Number, *BM* Bone Metastasis

### Risk model predictors

On multivariable logistic regression among development cohort in the risk model, rectal cancer, poorly-undifferentiation, signet-ring cell carcinoma, CEA positive, N1/N2 stages, brain metastasis, liver metastasis and lung metastasis were significantly associated with higher risk of developing BM, which were used to develop the risk model of predicting BM. The details were shown in Table 3.

**Table 3:** Risk factors for bone metastasis of colorectal cancer patients in multivariable logistic regression

Variable	Multivariable analysis			
	OR (95% CI)	*P* Value	Beta cofficients (β)	Scores
Tumor location				
Colon	1 [Reference]			
Rectum	1.960 (1.412–2.720)	0.000	0.673	2
Grade				
Well-Moderate differentiation	1 [Reference]			
Poorly-Undifferentiation	1.686 (1.224–2.323)	0.001	0.523	2
Histological type				
Adenocarcinoma	1 [Reference]			
Signet-ring cell carcinoma	4.904 (2.789–8.623)	0.000	1.590	4
CEA				
(−)	1 [Reference]			
(+)	2.067 (1.411–3.027)	0.000	0.726	2
N Stage				
N0	1 [Reference]			
N1/N2	1.471 (1.039–2.082)	0.029	0.386	1
Brain metastasis				
No	1 [Reference]			
Yes	13.602 (5.774–32.045)	0.000	2.610	6
Liver metastasis				
No	1 [Reference]			
Yes	8.378 (5.893–11.910)	0.000	2.126	5
Lung metastasis				
No	1 [Reference]			
Yes	5.563 (4.027–7.684)	0.000	1.716	4

*OR* odds ratio, *CI* confidence interval

### Risk scores

According to the criteria for assigning point values as previously described, the scores for each significant variable in the final risk model were calculated and presented in Table 3. The total maximal risk point value would be assigned 26 for one patient. In our study, total scores ranged from 0 to 20 in both development cohort and validation cohort.

### Model accuracy and validity

**Fig. 2:**
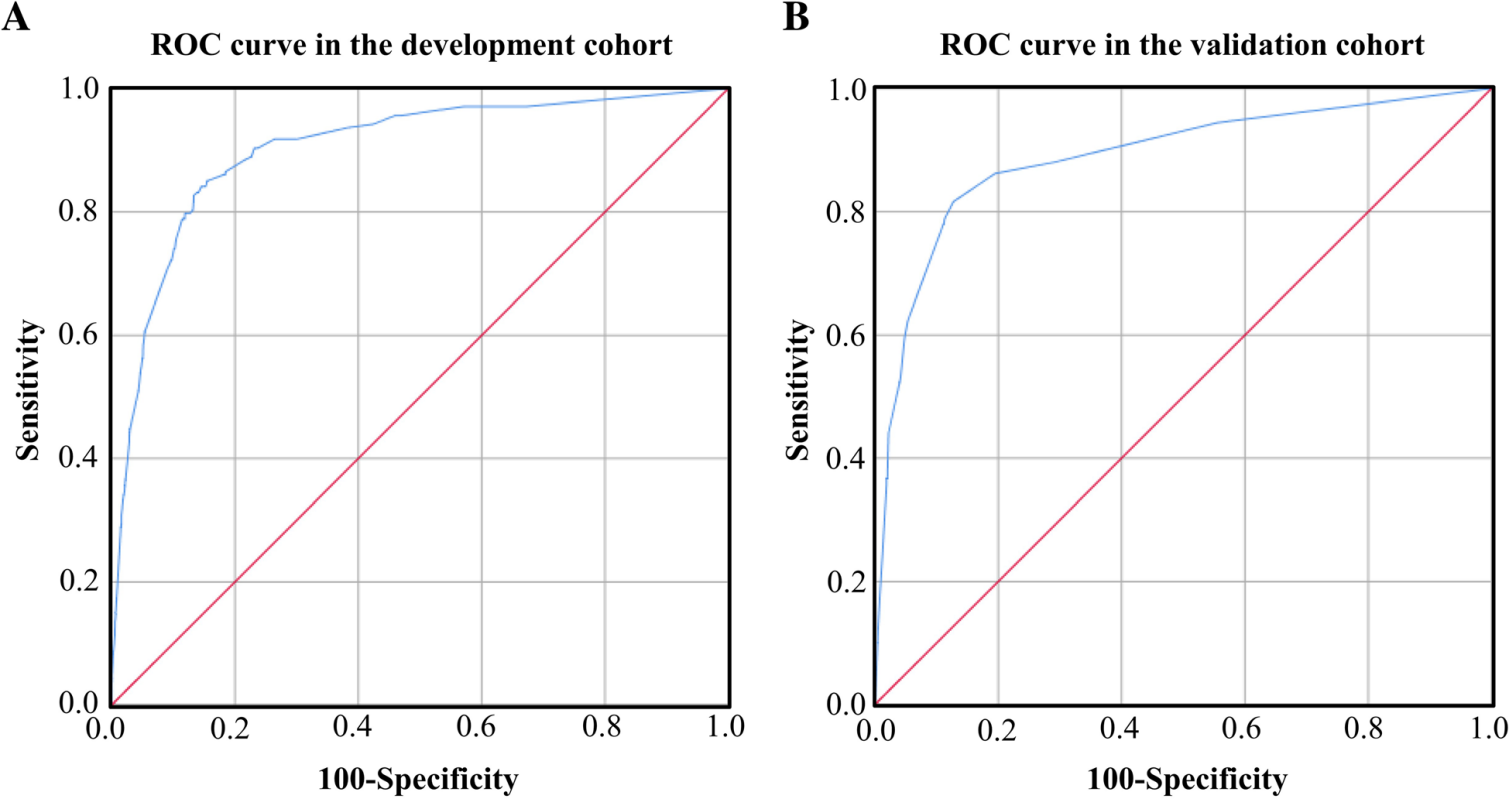
**a**. ROC curve in the development cohort; **b**. ROC curve in the validation cohort

**Fig. 3:**
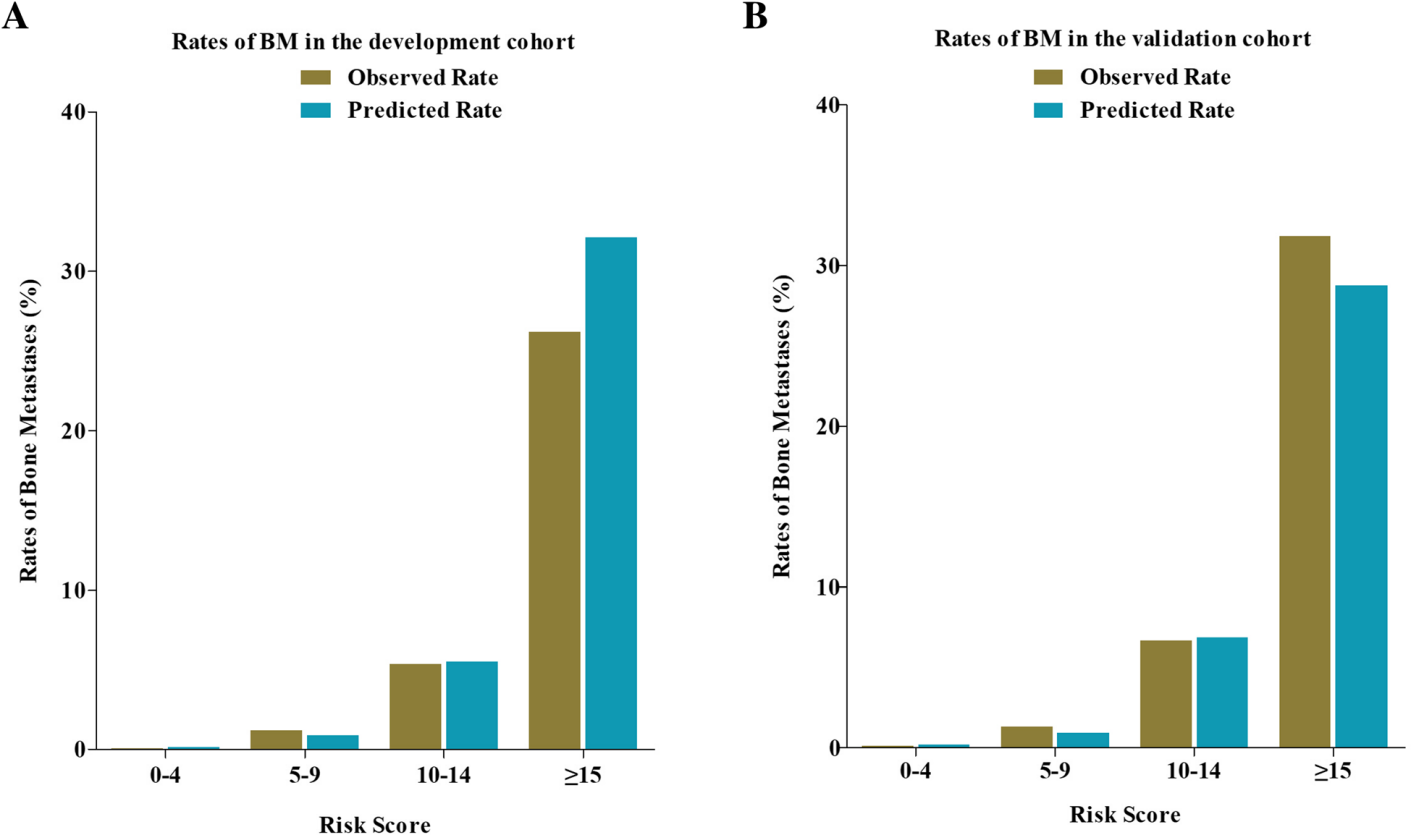
**a**. The predicted and observed rates of BM in the development cohort; **b**. The predicted and observed rates of BM in the validation cohort

The AUROC for the risk model was 0.903 in development cohort and 0.889 in validation cohort, which suggested very good discriminations of this risk model to accurately identify the risk of developing BM for CRC patients (Fig. 2). Then we used the Euclideans’s index to screen out the optimal cut off with 0.851 sensitivity and 0.845 specificity in development cohort, while 0.817 and 0.873 respectively in validation cohort. Risk stratification of BM was performed based on the risk scores. Patients with total score of 0–4, 5–9, 10–14, ≥15 points were respectively classified into very low risk group, low risk group, medium risk group and high risk group. Then, the observed and predicted rates of BM were compared in development cohort and validation cohort by four groups. The predicted rates of synchronous BM for each patient were calculated from the risk model estimates. In the very low risk patients, the predicted rates of BM were 0.13% in development cohort and 0.19% in validation corhort, as the observed rates were 0.10 and 0.13% respectively. In the high risk group, the predicted rates of BM obviously increased to 30.53% in development cohort and 31.87% in validation cohort, and the corresponding observed rates increased to 28.57 and 30.77% respectively. Figure 3 displayed the predicted and observed rates of BM in four groups by total risk score.

## Discussion

Currently, CRC is a major cause of morbidity and mortality globally [1]. Liver and lung metastasis frequently occur in newly diagnosed CRC, which are routinely recommended to be identified by systematic body examination during the primary diagnosis of CRC [6]. Here we found the proportion of BM incidences in CRC patients in our study is much less than the national reported proportion of BM incidences in CRC patients [3]. Apart from the unrepresentative sample of the overall population in the national findings, this can be due to the rare incidence of synchronous BM recorded in the SEER database instead of metachronous BM seen through several decades in the national study. Synchronous BM diagnosed in CRC patients is very rare, which therefore lead to ignorance of this special clinical entity [15]. However, with the increased overall survival of CRC patients, the incidence of BM from CRC has been on the rising during the course of CRC [4]. Therefore, early identification of synchronous BM during the primary diagnosis of CRC will contribute to the accurate staging and improvement of prognosis for CRC patients. And in current clinical practice, the diagnosis of synchronous BM is mainly based on the clinical SREs, but the optimal theraputic opportunity for BM has already missed when SREs occur [8]. Therefore, early identification of synchronous BM during the primary diagnosis of CRC will also contribute to delay the progress of SREs and improve the quality of life of patients. To better address this issue, we used population-based database to generate a risk model based on clinical and tumor characteristics to predict the risk of synchronous BM in newly diagnosed CRC patients. The results showed that this risk model could precisely identified the synchronous BM from CRC patients with considerably high accuracy.

Previous studies have been reported identifying the risk factors of BM and developing risk models to predict BM after curative resection of CRC. Sun et al. evaluated 516 patients who received curative resection for CRC and found two independent risk factors contributing to BM during the follow-up, including lymph node involvement and tumor location [12]. Ang et al. have found that three independent risk factors of BM, including rectal cancer, lymph node metastasis and lung metastasis. A scoring system was then developed to predict BM based on these three risk factors, and CRC patient were divided into three groups with different risk of developing BM (1.5% vs. 6.6 and 10.5%, *P* < 0.001) [13].

In our study, we developed the first risk model to predict the probability of synchronous BM at primary CRC diagnosis. Here, we found that rectal cancer was an independent risk factor associated with synchronous BM, which accorded with research of other scholars [12, 13]. Rectal and colon cancers have differences of anatomical location and tissue sources from the beginning of embryonic development. There are some communicating branches between rectal vein and vertebral vein system, which probably is one of the mechanisms of BM [3]. It also be found that patients with poorly or undifferentiated tumor and signet-ring carcinoma were more prone to have BM. This might be because cancer cells have capability of invading surrounding tissues, capillaries and lymphatics with stronger growth potential to develop early metastasis [16]. The CEA in serum and lymph node metastasis would promote the infiltration and metastasis of CRC, leading to higher risk of synchronous BM in CRC patients [17, 18]. Besides, extraosseous metastasis could also increase the risk of synchronous BM. Thus, we use the above significant risk factors to develop the risk model to predict BM. Furthermore, this risk model showed good discriminations with the AUROC being 0.903 in the development and 0.889 in the validation cohort, which could be accurate to identify the newly diagnosed CRC patients who should require more comprehensive assessment to detect potential BM.

The strengths in this study include large sample size from SEER database and good accuracy for predicting synchronous BM in CRC patients. Furthermore, the prediction model consist of accessibly clinical and tumor characteristics, which suggested that this model should be more clinically acceptable. However, due to all patients in this study only representing the population in US, this risk model has the limitation in its widespread use, and the external validation involving the population from other countries should be needed to further confirm the accuracy of this risk model. Another limitation of this study is the lack of biological markers, which could be incorporated into this statistical model to improve the accuracy and its usefulness. Despite these limitations, this risk model remains a accurate and valuable clinical tool to predict the risk of developing synchronous BM in newly diagnosed CRC patients.

## Conclusions

Although the patients with BM only accounted for a small proportion in CRC patients, early detection of synchronous BM with routine screening at the primary diagnose of CRC would be beneficial for high risk patients. Here, this clinical prediction model present high accuracy to identify the newly diagnosed CRC patients with high risk of BM and provide more individualized decision making to this group of patients.

## Data Availability

The database used and/or analyzed during the current study are available from the corresponding author on reasonable request.

## Abbreviations

AUROC: Areas under the receiver operating curve
BM: Bone metastasis
CEA: Carcinoembryonic antigen
CI: Confidence interval
CRC: Colorectal cancer
N: Number
NA: Not available
OR: Odds ratio
ROC: Receiver operating curve
SEER: Surveillance, epidemiology, and end results
SREs: Skeletal-related events
